# GalenOWL: Ontology-based drug recommendations discovery

**DOI:** 10.1186/2041-1480-3-14

**Published:** 2012-12-20

**Authors:** Charalampos Doulaverakis, George Nikolaidis, Athanasios Kleontas, Ioannis Kompatsiaris

**Affiliations:** 1 Centre for Research and Technology Hellas, Information Technologies Institute, Thessaloniki, Greece; 2, Ergobyte S.A., Thessaloniki, Greece; 3, Theagenio Cancer Hospital, Thessaloniki, Greece

## Abstract

**Background:**

Identification of drug-drug and drug-diseases interactions can pose a difficult problem to cope with, as the increasingly large number of available drugs coupled with the ongoing research activities in the pharmaceutical domain, make the task of discovering relevant information difficult. Although international standards, such as the ICD-10 classification and the UNII registration, have been developed in order to enable efficient knowledge sharing, medical staff needs to be constantly updated in order to effectively discover drug interactions before prescription. The use of Semantic Web technologies has been proposed in earlier works, in order to tackle this problem.

**Results:**

This work presents a semantic-enabled online service, named GalenOWL, capable of offering real time drug-drug and drug-diseases interaction discovery. For enabling this kind of service, medical information and terminology had to be translated to ontological terms and be appropriately coupled with medical knowledge of the field. International standards such as the aforementioned ICD-10 and UNII, provide the backbone of the common representation of medical data, while the medical knowledge of drug interactions is represented by a rule base which makes use of the aforementioned standards. Details of the system architecture are presented while also giving an outline of the difficulties that had to be overcome. A comparison of the developed ontology-based system with a similar system developed using a traditional business logic rule engine is performed, giving insights on the advantages and drawbacks of both implementations.

**Conclusions:**

The use of Semantic Web technologies has been found to be a good match for developing drug recommendation systems. Ontologies can effectively encapsulate medical knowledge and rule-based reasoning can capture and encode the drug interactions knowledge.

## Background

One of the health sectors where intelligent information management and information sharing compose valuable preconditions for the delivery of top quality services, is personalized drug prescription. This is more evident in cases where more than one drug is required to be prescribed, a situation which is not uncommon, as drug interactions may appear. The problem is magnified by the wide range of available drug substances in combination with the various excipients in which the former are present. Another factor that makes drug prescription a complex task is the complexity that characterizes the definition of possible interactions or contraindications due to the large number of parameters that are implicated.

Indicatively, it is mentioned that, according to statistics, men over 55 years old, daily consume four different medicines on average and the reactions that can occur due to combined prescription are difficult to predict. As an example, the substance *Donepezil* (ATC code: N06DA02) which is prescribed for the treatment of Alzheimer’s disease interacts with 9 other substances and 3 other diseases. If it is taken into account that there exist more than 18,000 pharmaceutical substances, including their excipients, then it is clear that the continuous update of health care professionals is remarkably hard. Over this, the extensive literature makes discovery of relevant information a time consuming and difficult process, while the different terminologies that appear between sources add more burden on the efforts of medical professionals to study available information.

Semantic Web technologies can play an important role in the structural organization of the available medical information in a manner which will enable efficient discovery and access. Semantic Web has already infiltrated in the public health sector [1] as a mean for representation of available knowledge or through the utilization of reasoning methodologies for automating procedures such as diagnosis, data classification, medical record consolidation, etc.

More specifically, with the use of ontology languages such as OWL, a rather large amount of biomedical ontologies have been developed among them ontologies of large size such as the Biological Pathways Exchange (BioPax) [2], the GALEN ontology [3], the Foundational Model of Anatomy (FMA) [4] as well as the Gene Ontology [5] and SNOMED CT [6].

The use of OWL for the expression and representation of the aforementioned ontologies, apart from the benefits regarding knowledge reuse and sharing that come from the use of a standardized language, revealed the benefits of semantic reasoning. The validation of the ontologies using OWL reasoning engines revealed important modelling failures but also a large number of subsumption relations that were missing from the initial requirements and not locating them would mean the loss of information in patient management systems.

Research projects funded for enabling Semantic Web technologies in the diagnosis and therapeutic procedures exist such as TUMOR [7], REMINE [8] and PSIP [9], with the latter aiming at reducing drug prescription adverse effects through data mining and semantic interpretation of a patient’s medical record. Other projects like NeOn [10] and Active Semantic Documents [11] employ ontologies in daily medical practice. Despite the research activity, there have been few proposals for a systematic development of a semantic knowledge base which will aid physicians when prescribing drugs. [12] describes a framework for information integration for drug safety determination using ontologies and in [13] authors suggest an approach to semantically annotate Electronic Discharge Summaries in order to provide decision support to physicians.

The paper presents GalenOWL, a semantic-enabled system for discovering drug recommendations and interactions. GalenOWL makes use of established and standardized medical terminologies together with a rich knowledge base of drug-drug and drug-diseases interactions expressed as rules and OWL axioms. GalenOWL is implemented as an online service having in mind, both completeness of results and responsiveness in query answering.

## Methods

### Development

The stimulus for developing GalenOWL was given by an already available market product. The GALINOS drug guide, available at *http://www.galinos.gr* in Greek, is an online service where a user can query the drug database and get information on available drugs that are found in the market, e.g. indications, recommended dosage, excipients, interactions, adverse effects, etc, where all the latter are related to the drugs active substances. All the above were mined after extensive research in the literature and of available documents such as Summary of Product Characteristics (SPC) and Patient information leaflets (PIL). For enabling this kind of functionality, GALINOS employs international medical standards which allow a unique identification of diseases and substances. It was evident that the knowledge integrated in the service could be used in order to develop an intelligent system for offering drug recommendations.

**Figure 1:**
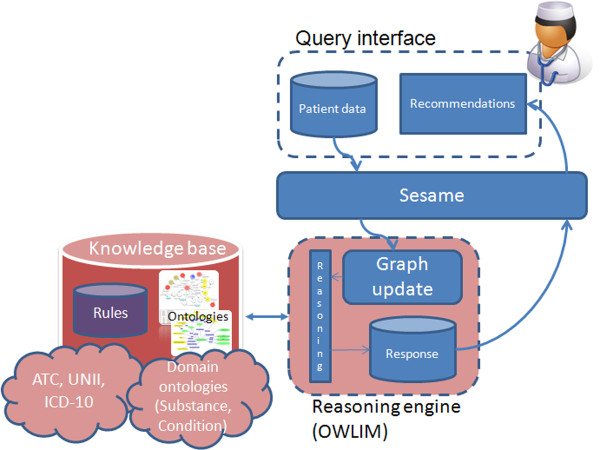
**GalenOWL usage.** Usage of the GalenOWL system showing processing and information flow.

GalenOWL architecture can be seen in Figure 1. The user issues queries to the system in order to find drug indications and contraindications that match patient data. These data populate the knowledge base and rule-based reasoning is performed. The reasoning engine makes use of the medical ontologies and the rule base for drug recommendations and a list of the drug recommendations (indications and contraindications) is returned by the engine. GalenOWL is novel in its field as, to the authors knowledge, there are no commercial systems that offer drug-diseases interactions. Systems that offer drug-drug interactions are available such as the one offered by Drugs.com^a^.

In GalenOWL, a drug-disease interaction, i.e. an (adverse) interaction between a drug and a disease, is defined if one of the following 3 facts hold: a) a drug that is administered for a particular disease may affect the progress of another disease. For example a drug for treating an upper respiratory infection should not be prescribed to a patient with renal failure if that drug aggravates renal function, b) the existence of a disease may affect the pharmacokinetics of a drug, e.g. a disease could increase a drug’s catabolism, thus reducing its effectiveness, or it could reduce its metabolism, thus causing accumulation of the drug in the body and lead to toxic reactions, c) precise contraindications where a drug cannot be prescribed for a patient that suffers from a specific condition, e.g. an anticoagulant drug cannot be prescribed to a patient that shows signs of internal bleeding.

### Development details

For the OWL/XML serialization, the Jena Semantic Web Framework^b^ was used. The OWL reasoner which provided the drug recommendations is OWLIM-Lite^c^ together with Sesame^d^ for providing the REST interface, the RDF data access and management platform and the SPARQL query interpretation layer. OWLIM was chosen as it has been found as one of the most efficient OWL reasoners [14,15].

### International standards ontologies

In order to provide such a service, coupling of Semantic Web and medical terminologies was needed. GalenOWL is built on top of OWL ontologies which express international standards of medical terminology in order to process requests for drug recommendations. The following terminologies are expressed as OWL ontologies: 

• **ICD-10**:The World Health Organization classification of diseases. It is used in GalenOWL for unique identification of diseases thus uniquely identifying drug indications and contraindications related to diseases.

• **UNII**: Unique Ingredient Identifier. Used for the identification of active ingredients found in drugs. In GalenOWL it is used for uniquely identifying drug indications and contraindications related to ingredients.

• **ATC**: The Anatomical Therapeutic Chemical Classification is used for the classification of drugs. In GalenOWL it is used in similar fashion to UNII.

Each code in the above encodings is expressed as an OWL class.

### Domain ontologies

Besides these international standards, two more classifications are expressed in OWL in order to make easier use of the system:*Substance*: As the use of encodings for drug ingredients is not convenient for humans, the identification of active substances is done using its common name references in medical bibliography. These names come from international standards such as the International Nonproprietary Names (INN) and others such as USAN (United States Adopted Name) or BAN (British Approved Name). Members of this identification list are substances such as *acetazolamide* or *isradipine*. In addition, substances correspond to ATC codes and this is captured in the ontology through class equivalence such that for example *acetazolamide* ≡ S01EC01.

*Condition*: As certain “groups” of substances and/or diseases are frequently present in drug interactions and these groups are not recorded explicitly in any standardized classification, it is more convenient for medical use to specify these custom groups. These often used groups are termed “conditions” in GalenOWL and are defined by medical experts. An example of such condition is *barbituratesdrugs* which is defined as

$$\begin{array}{c} \text{barbituratesdrugs}=\text{a/N01AF}|\text{a/N01AG}|\text{a/N03AA}|\\ \;\text{a/N05CA}|\text{a/N05CB}|\text{a/N05CX} \end{array}$$

where “a/” stands for ATC code and “|” stands for “or”. So any member of these premises is also a member of *barbituratesdrugs*. In addition a condition can appear as a premise in other condition definitions. So the condition

$$\begin{array}{c} \text{hemorrhage-postoperative}=\text{c/hemorrhage-nos}\;\&\\ \;\text{c/surgical-dental-procedures} \end{array}$$

is satisfied when two other Conditions (denoted by “c/”) are satisfied simultaneously (denoted by “&” which stands for “and”). It is evident that conditions can be effectively expressed in OWL as defined classes. In the above examples, “a/” or “c/” would represent the namespace of the ontology and “|” or “&” would represent the union or intersection of classes respectively. Using DL notation the above classes are represented as

$$\begin{array}{c} \text{barbituratesdrugs}\equiv N01\text{AF}\;ப\;N01\text{AG}\;ப\;N03\text{AA}\;ப\\ \;N05\text{CA}\;ப\;N05\text{CB}\;ப\;N05\text{CX}\; \end{array}$$

and

$$\begin{array}{c} \text{hemorrhage-postoperative}\equiv \text{hemorrhage-nos}\;⊓\\ \;\text{surgical-dental-procedures} \end{array}$$

In order to automate the definition of the Conditions ontology, a parser was developed to express the conditions from the custom format explained above to OWL/XML notation.

**Figure 2:**
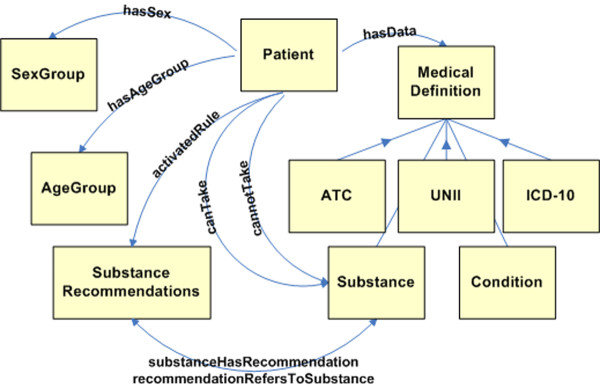
**GalenOWL core ontology.** Diagram displaying the main classes of the GalenOWL ontology along with their properties.

All the above mentioned ontologies were imported from the GalenOWL core ontology depicted in Figure 2. Additionally, *Patient* is the class for patient instances. Patient instances are related with the *MedicalDefinitions* and with *AgeGroup* and *SexGroup* through the *hasAgeGroup* and *hasSexGroup* properties respectively.

### Rule base

After the definition of the domain ontologies and the core ontology, an appropriate rule base for indications and contraindications was defined. The rules are expressed in a custom language similar to the Conditions of the previous subsection. These rules however usually have a more complex syntax. An example rule for the indication of *rimonabant* is defined as

$$\begin{array}{c} \text{rimonabant}=i/E65-E68\;\&(i/E11|i/E78) \end{array}$$

where “i/” stands for ICD-10 code and it reads as: *rimonabant* is indicated in cases where E65-E68 **and**, E11 or E78, diseases are present. In DL this is represented as “***rimonabant***≡ E65-E68 ⊓ (E11 ⊔ E78)”.

Due to GalenOWL being developed using OWLIM-Lite, the above expression had to be expressed in the OWLIM custom rule language. “or” could not be expressed in a rule, so two different rules were generated for *rimonabant*. To make things more complicated, drug indications also depend on the patient’s sex and age. In the above example, *rimonabant* is prescribed only for adults or elder patients so this also had to be encoded in the rules. As a result, 4 rules were built for *rimonabant* indication (gl: stands for the GalenOWL namespace), i.e. 

1) *Patient*(?*p*), *hasData*(?*p*, icd:E65-E68), *hasData*(?*p*, icd:E11), *hasAgeGroup*(?*p*, gl:adult) → *canTake*(?*p*, sub:rimonabant)

2) *Patient*(?*p*), *hasData*(?*p*, icd:E65-E68), *hasData*(?*p*, icd:E78), *hasAgeGroup*(?*p*, gl:adult) → *canTake*(?*p*, sub:rimonabant)

3) *Patient*(?*p*), *hasData*(?*p*, icd:E65-E68), *hasData*(?*p*, icd:E11), *hasAgeGroup*(?*p*, gl:elder) → *canTake*(?*p*, sub:rimonabant)

4) *Patient*(?*p*), *hasData*(?*p*, icd:E65-E68), *hasData*(?*p*, icd:E78), *hasAgeGroup*(?*p*, gl:elder) → *canTake*(?*p*, sub:rimonabant)

Of course, indication rules have no limitation in the premises separated by “or” which can lead to a very big rule expansion. As an example, *buspirone* has 13 premises separated with “or” which leads to 13 different rules. In the current version of GalenOWL 1342 substance indications/contraindications were expressed using 9266 rules. A parser similar to the one developed for Conditions was used in order to express the indications in the OWLIM custom rule language. Although the rule base is quite large in size, OWLIM’s sophisticated indexing structure and rule engine was quite fast in evaluation of rule activation.

Another rule that was necessary is one that would evaluate the conflicts between indications and contraindications regarding a patient’s conditions. For example, a substance could be indicated in the case of a specific disease but the same substance could also be contraindicated in the case of another disease. This will result in substance appear both in the indications and contraindications. Clearly this substance should be excluded from the recommended prescription. In order to discover these substances, a special rule was expressed as “*canTake*(?*p*, ?*s*), *cannotTake*(?*p*, ?*s*) →* hasSubstanceConflict*(?*p*, ?*s*)” and was incorporated in the rule base.

Finally, for each rule an instance under *Indications* or *ContraIndication* class (both subclasses of *SubstanceRecommendations*) is created and the property *hasTextualRepresentation* is set to the original textual representation of the rule. This is used in order to provide tracing in rule matching so that for each rule that is activated the property *activatedRule(patient, recommendation)* is materialized. These relations are depicted in Figure 2. In the GalenOWL ontologies a total of 28,867 named classes were defined. Table 1 gives information about the size of the ontologies imported in GalenOWL.

**Table 1:** GalenOWL ontology metrics

**Ontology**	**Number of classes**
ATC	5596 (primitive)
ICD-10	12108 (primitive)
UNII	6759 (primitive)
Substances	2823 (primitive)
Conditions	68 (defined)
Indications-ContraIndications	1342 (primitive)
**Total**	28867

Number of primitive and defined classes in the GalenOWL ontologies

### Interface and querying

**Figure 3:**
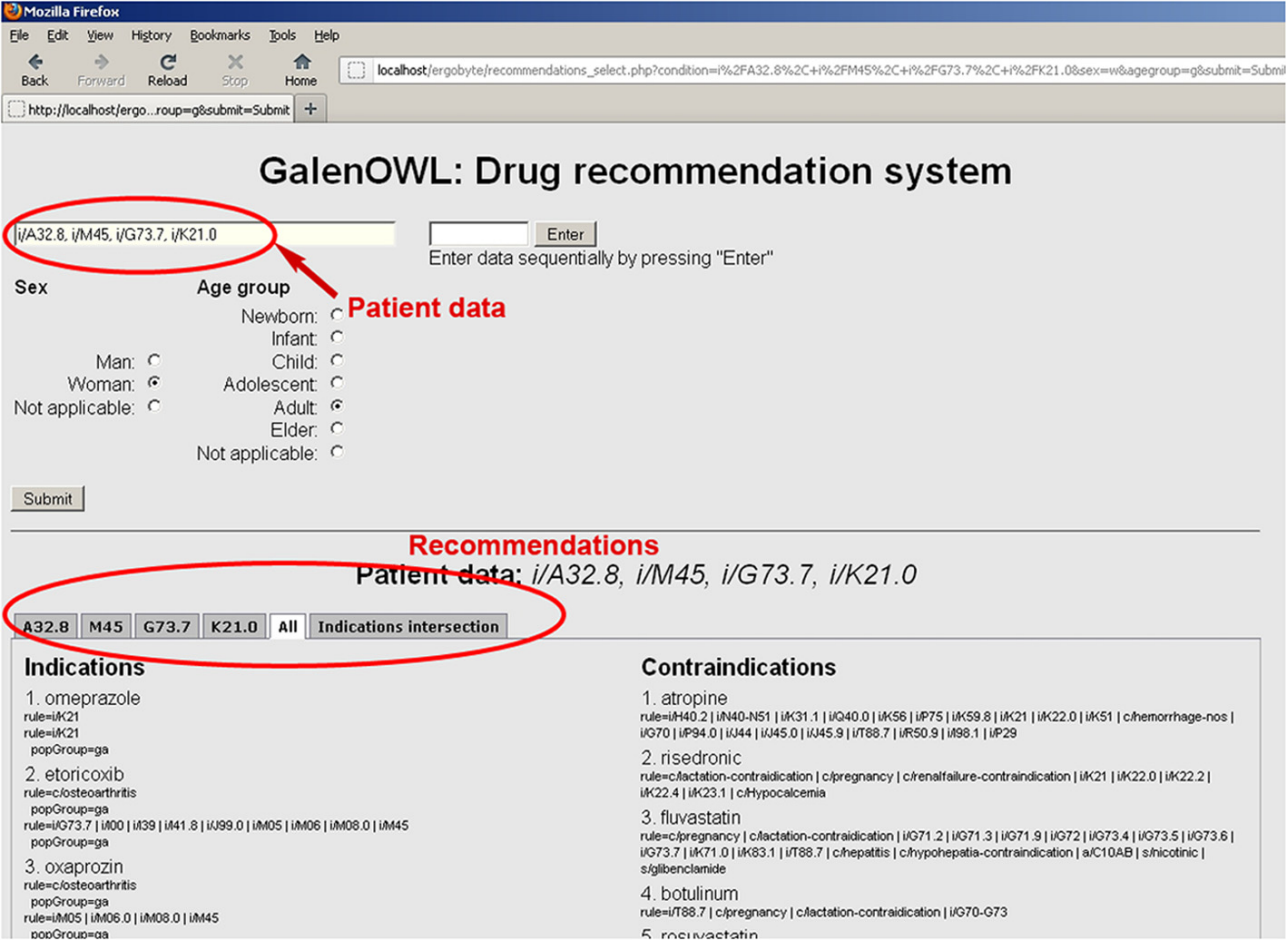
**User interface and results.** Snapshot of the prototype user interface.

The interface to the system is depicted in Figure 3. As the focus on the system was on the functionalities that can be provided and on its capabilities, the design of the interface may lack in aesthetic design nevertheless it provides all the information that are returned from the system in a rather easy to use layout.

Patient data regarding diseases, allergies, population group and current medication are entered sequentially using the form. After all data are entered, the user submits all information to the system in order to be inserted in the knowledge base as an RDF graph which represents patient data. During insert, all inferences using OWL reasoning and rule execution are performed and are also stored in the knowledge base thus making query answering faster as no complex inference is performed during query time. Recommendations lists from GalenOWL are retrieved using separate SPARQL queries (querying for indications, contraindications and conflicts) which are sent from the user interface to the Sesame server through REST.

In order to provide an overall view of the drug recommendations that are returned by GalenOWL the following sequence of actions is performed: Each patient data (disease, allergy, current medication) that is in the list is inserted separately and inference is performed. This is done so that the user can have a list of recommendations that is due to each data separately. In a final step all data are entered simultaneously so that recommendations that are valid for all patient’s data are evaluated. All recommendations are separated in 4 groups, the indications list, the contraindications list, the conflicts between indications and contraindications, i.e. substances that appear both in indications and contraindications which can be expressed as (*indications*∩*contraindications*), and a cleared list where only indications that do not appear in contraindications are present. This list actually represents the valid recommendations of the system for the patient’s prescription, i.e. *indications*∖ (*indications*∩*contraindications*). Results lists are separated in tabs and each tab corresponds to one of the sequential steps described above. For easing the burden to the query engine, the last set of valid recommendations is composed programmatically from the user interface by comparing and combining the indications and conflicts lists.

### Business logic implementation

For having a broader view of GalenOWL’s performance, a similar system has been developed using standard business logic programming technologies. This system has been termed GalenDrools as in its core for drug recommendations lies the Drools rule engine [16], which is an open source and efficient framework for business logic integration.

To give a brief description of GalenDrools implementation, ICD-10, ATC and UNII encodings as well as Substance and Conditions, are stored in a database. For building the rule base the indications/contraindications rules are parsed and translated to the Drools rule language (DRL). When premises for ICD-10 or ATC classification codes are present in the rule body, the latter is automatically populated with upper level codes of the classification, in a manner similar to the Class/SubClass relation in ontologies. One more different aspect of GalenDrools architecture is the way that Conditions are handled. While in GalenOWL Conditions are translated into OWL defined classes, here each condition that appears in a rule is recursively expanded to its primitive elements, i.e. ICD-10, ATC, UNII or Substance codes. As an example, let us assume a rule for prescribing the substance *mefenamic* where it states:

***mefenamic*** = c/arthropathy-inflammatory-indication | i/N94.4 where “arthropathy-inflammatory-indication = i/M05-M14 | i/M15-M19”. The DRL rule would have to be expanded in order to take into account the Condition definition and the class relationships. As such, it would be expressed as: 

RULE “example1”

WHEN

p: Patient((data==M05-M14) || (data==M15-M19) ||

(data==M00-M99) || (data==N00-N99) ||

(data==N80-N98) || (data==N94) || (data==N94.4))

THEN

p.prescription = mefenamic

END

In the above rule, one can notice how the expansion of both the Condition definition and of the class/subclass relations is performed. This knowledge, although it is already stored in the database, it has to be separately declared inside the rule expression.

When requesting drug recommendations, patient data are inserted as facts in the Drools truth maintenance table and rule execution is initiated. These facts actually correspond to the database IDs of the ICD-10, ATC, UNII and Substance codes which makes rule matching quite fast.

## Results and discussion

### GalenOWL performance

In order to verify GalenOWL’s functionality in terms of results completeness in drug recommendations, a series of random queries regarding patient data (diseases, current medication, population groups, etc.) were submitted to the system and the results were evaluated by a medical expert. The analysis concluded that the results were as expected and all patient’s conditions were taken into account. A series of tests in order to determine initialization time, memory consumption and query response time of GalenOWL have been performed. These values are reported in the first row of Table 2 where promising results are reported especially in query response time which is kept at a satisfying 16 ms average time. The initialization phase, which included compilation of the rule base and loading of the ontology in the main memory took 148 seconds which is reasonable if the large volume of the knowledge base (ontologies plus rules) is taken into account and the fact that this is a one time task executed only during initialization. What is less than ideal is the memory consumption after the initialization phase which stays constant at around 649 MB and takes up a fairly big amount of system resources which is undesirable in a production environment.

**Table 2:** GalenOWL vs GalenDrools

	**Initialization**	**Memory**	**Query time**
GalenOWL	148 s	649 MB	16 ms
GalenDrools	41 s	74 MB	5 ms

GalenOWL performance compared to a similar system developed in Drools (GalenDrools).

### GalenOWL compared to GalenDrools

As it is depicted in Table 2, a direct comparison between GalenOWL and GalenDrools reveals that in almost all aspects the business logic implementation of the drug recommendations system outperforms the semantic-enabled implementation by an order of magnitude. Initialization of GalenOWL takes more time as the rule base has to be compiled and all inferences computed during the ontology loading. Memory consumption is high as the whole ontology and rule base have to be loaded in memory. On the contrary, in GalenDrools the initialization phase includes only the compilation of the rule base which is the only structure stored in memory thus making it more efficient both in startup time and in memory consumption. Regarding query response time, in GalenOWL when a new patient instance is inserted, inference is performed which leads to increased response time compared to GalenDrools where simple rule matching is performed.

On the other hand, although inference adds burden and overhead to query response, it actually makes development of the system easier. In the business logic implementation both the subclass relations and the expansion of Conditions had to be implemented programmatically and encoded in the rule body, a process which requires effort and increases the possibility to induce errors, but it also combines medical knowledge, e.g. from Conditions definition, with drug administration rules. In contrast, in the OWL-based implementation, issues such as hierarchical class relationships and derived consequences such as class membership, were automatically identified by the reasoning engine and medical knowledge is defined solely in the ontology. The pharmaceutical knowledge is separately expressed in the rules. Additionally, the GalenOWL implementation promotes knowledge sharing as the ontologies that were developed can be accessed by using Semantic Web technologies, e.g. they can be exposed as SPARQL endpoints and queries can be issued. In GalenDrools, since a database back-end is used for storing knowledge, exposing this information is not straightforward and queries to the database have to follow the schema, which is not easy to expose. Finally, regarding the rule definitions, the GalenOWL approach directly express the pharmaceutical knowledge using rules and the latter can be directly loaded to any ontology reasoner. In the GalenDrools approach, the rules have to be processed so that the entity relationships and hierarchies are materialized inside the rule body. This makes rule definition specific to the business rule engine which will be used. Taking into account all the above, we can conclude that ontologies provide a good match for such systems as they can effectively integrate knowledge and inherently make it available for further use. Table 3 summarizes the qualitative comparison between the two approaches.

**Table 3:** Qualitative comparison between GalenOWL and GalenDrools

	**GalenOWL**	**GalenDrools**
Structured knowledge representation	Yes, ontology based.	Partial, relational DB.
Medical knowledge integration and reusability	Hierarchical class relationships (ICD10, UNII, ATC) and definition of Conditions are expressed using OWL expressivity. They can be utilized by any OWL reasoner.	ATC, UNII, ICD10 entities relationships and Conditions are materialized inside rule expressions. Materialization is specific to the rule language used.
Knowledge sharing	Ontology can be published and accessed through SW technologies, e.g. as a SPARQL endpoint.	Queries to DB have to follow the DB schema.
Rule expression	Rules for drug recommendations directly express pharmaceutical knowledge and can be immediately loaded to a reasoner.	Rules express pharmaceutical knowledge but have to be post processed, in order to materialize entities relationships before loading them to the rule engine.

Table summarizing the major qualitative differences between GalenOWL and GalenDrools.

The efficiency of production rule engines has already been utilized in Semantic Web literature. In [17] the authors use the CLIPS rule engine as an OWL reasoner after transforming the OWL ontology to the COOL object oriented language of CLIPS. However ontology management and querying become demanding tasks. In OWLJessKB [18] the Jess rule engine is used for OWL reasoning where the RDF triples are inserted as facts and OWL entailments are materialized using production rules. This approach though suffers from memory limitations. It should be noted that business rule engines have been around for much longer time than OWL reasoners and they are aimed at much larger audience than Semantic Web technologies. This alone corresponds to a much larger community contributing to frameworks like Drools. These two facts can account for the exceptional performance that these systems exhibit. The authors believe that as the Semantic Web community grows larger, more frameworks that will be able to compete traditional rule engines will be made available. OWLIM is an example of an efficient reasoning engine and up to now several other reasoners are claiming increased performance such as HermiT [19] and TrOWL [20].

## Conclusion

In this paper a drug recommendation system based on Semantic Web technologies, termed GalenOWL, was presented. It has been shown that OWL and Semantic Web technologies can provide a good match for drug recommendations as OWL is expressive enough to effectively encapsulate medical knowledge. Rule-based reasoning can model medical decision making and provide assistance to experts. A comparison of the semantic-enabled implementation to a traditional business logic implementation was presented. Although the latter has shown better performance in time and memory requirements, semantic technologies provide a better alternative for integrating knowledge in the system than simple rule engines.

Future work, apart from the expansion of the semantic rule base, will include prioritization of interactions so not all interactions have the same importance. Additional work will be directed to research oriented performance optimizations, such as context extraction from medical knowledge and from queries which will lead to modular ontologies, so that not to take into account the whole ontology during query time. This will result in less memory utilization and better query response times.

## Endnotes

^a^Drugs.com, http://www.drugs.com/drug_interactions.php. ^b^Jena Semantic WebFramework, http://incubator.apache.org/jena. ^c^OWLIM-Lite, http://www.ontotext.com/owlim. ^d^Sesame, http://www.openrdf.org.

## Competing interests

The authors declare that they have no competing interests.

## Author’s contributions

CD was the lead developer of GalenOWL and wrote major parts of the manuscript, GN was the lead developer of GalenDrools and contributed to the writing of the manuscript, AK contributed in the verification of the 2 systems and to the writing of the manuscript and IK had the scientific lead and contributed to the writing of the manuscript. All authors read and approved the final manuscript.
